# Unconditional and conditional standards for fetal abdominal circumference and estimated fetal weight in an ethnic Chinese population: a birth cohort study

**DOI:** 10.1186/s12884-015-0569-1

**Published:** 2015-06-25

**Authors:** Ying Xu, Ngee Lek, Yin Bun Cheung, Arijit Biswas, Lin Lin Su, Kenneth Y. C. Kwek, George S. H. Yeo, Shu-E Soh, Seang-Mei Saw, Peter D. Gluckman, Yap-Seng Chong

**Affiliations:** Duke-NUS Graduate Medical School, National University of Singapore, Singapore, Singapore; Department of Pediatrics, KK Women’s and Children’s Hospital, Singapore, Singapore; Department of International Health, University of Tampere, Tampere, Finland; Department of Obstetrics and Gynaecology, Yong Loo Lin School of Medicine, National University of Singapore, National University Health System, Singapore, Singapore; Division of Obstetrics and Gynaecology, KK Women’s and Children’s Hospital, Singapore, Singapore; Department of Paediatrics, Yong Loo Lin School of Medicine, National University of Singapore, National University Health System, Singapore, Singapore; Saw Swee Hock School of Public Health, Yong Loo Lin School of Medicine, National University of Singapore, Singapore, Singapore; Singapore Institute for Clinical Sciences, Agency for Science Technology and Research (A*STAR), Singapore, Singapore; Liggins Institute, University of Auckland, Auckland, New Zealand

**Keywords:** Abdominal circumference, Conditional standards, Estimated fetal weight, Fetal growth, Ultrasound

## Abstract

**Background:**

Diagnosis of intrauterine fetal growth restriction and prediction of small-for-gestation age are often based on fetal abdominal circumference or estimated fetal weight (EFW). The present study aims to create unconditional (cross-sectional) and conditional (longitudinal) standards of fetal abdominal circumference and EFW for use in an ethnic Chinese population.

**Methods:**

In the Growing Up in Singapore Towards healthy Outcome (GUSTO) birth cohort study in Singapore, fetal biometric measurements were obtained at enrolment to antenatal care (11-12 weeks) and up to three more time points during pregnancy. Singleton pregnancies with a healthy profile defined by maternal, pregnancy and fetal characteristics and birth outcomes were selected for this analysis. The Hadlock algorithm was used to calculate EFW. Mixed effects model was used to establish unconditional and conditional standards in z-scores and percentiles for both genders pooled and for each gender separately.

**Results:**

A total of 313 women were included, of whom 294 had 3 and 19 had 2 ultrasound scans other than the gestational age dating scan. Fetal abdominal circumference showed a roughly linear trajectory from 18 to 36 weeks of gestation, while EFW showed an accelerating trajectory. Gender differences were more pronounced in the 10^th^ percentile than the 50^th^ or 90^th^ percentiles. As compared to other published charts, this population showed growth trajectories that started low but caught up at later gestations.

**Conclusions:**

Unconditional and conditional standards for monitoring fetal size and fetal growth in terms of abdominal circumference and EFW are available for this ethnic-Chinese population. Electronic spreadsheets are provided for their implementation.

**Electronic supplementary material:**

The online version of this article (doi:10.1186/s12884-015-0569-1) contains supplementary material, which is available to authorized users.

## Background

Intrauterine fetal growth restriction (IUGR) and small-for-gestation age (SGA) are associated with elevated risk of adverse birth outcomes in the short-term and cardio-metabolic diseases in the long-term [1–4]. Early and accurate diagnosis of IUGR and prediction of SGA may allow timely interventions to minimise adverse perinatal, childhood and adult health outcomes.

Ultrasound surveillance of biometric parameters, including biparietal diameter (BPD), head circumference (HC), abdominal circumference (AC) and femur length (FL), is an integral part of antenatal care. Estimated fetal weight (EFW) is calculated using the Hadlock algorithm based on ultrasound measurements of AC, BPD, HC and FL. A recent study showed that AC gave larger area under receiver operating characteristic curve than EFW and HC in predicting SGA [5], but two other studies did not [6, 7]. Recently published and updated clinical practice guidelines in various countries recommend using AC or EFW below the 10th percentile for gestational age (GA) to diagnose IUGR and/or predict SGA [8–11].

Most fetal biometry norms are derived from Caucasian populations in Europe and northern America despites calls for ethnic-specific norms for use in other populations [12–19]. The INTERGROWTH-21st Project maintained that skeletal size parameters are good options for cross-country comparison of fetal growth [20]. However, current clinical practice requires AC and EFW instead of skeletal size parameters. The paucity of ethnic-specific standards has prompted the development of a generic “global” fetal growth reference for fetal weight, which is based on an assumption of proportionality [21]. This has been lauded as an interim step towards better customized fetal growth charts [22].

Most standards are cross-sectional, or unconditional. They are more appropriate for the quantification of fetal “size” than fetal “growth” [23, 24]. Longitudinal standards, also called conditional standards, take previous assessment result into account and is suitable for studies of growth. It has been hypothesized that conditional standards are more powerful in identifying fetal growth abnormalities than unconditional standards [25].

Taking these issues into considerations, this study aimed to develop unconditional and conditional AC and EFW standards for 18 to 36 weeks of gestation for use in an ethnic-Chinese population with singleton pregnancy.

## Methods

### Study design and participants

Singapore is a city-state in South-East Asia, with 74.1 % of its population being ethnic Chinese in 2010 [26]. The Growing Up in Singapore Towards healthy Outcomes (GUSTO) study is a mother-offspring cohort study. Details of the study have been reported previously [27]. Briefly, pregnant women aged 18 years or above who had their first trimester antenatal dating ultrasound scan at the maternal units of Singapore’s two major public hospitals (KK Women’s and Children’s Hospital and National University Hospital) between June 2009 and September 2010 were recruited. This study was approved by both the National Healthcare Group Domain Specific Review Board and the SingHealth Centralized Institutional Review Board. Written informed consent was obtained from each participating pregnant woman in early pregnancy.

Singletons, conceived naturally, whose mother and father were both ethnic Chinese were included in the present analyses. For the purpose of developing standards, we selected a “healthy” group of participants. Mothers with the following characteristics were excluded: pre-pregnancy diabetes, pre-pregnancy hypertension, previous miscarriage, previous still birth, smoking during pregnancy, alcoholic consumption during pregnancy, self-reported pre-pregnancy body mass index (BMI) <17 kg/m^2^ or ≥27 kg/m^2^, pre-eclampsia and/or pregnancy-induced hypertension, and/or diabetes diagnosed by oral glucose tolerance test between 26 and 28 weeks of gestation according to the WHO criterion [28] or fasting plasma glucose levels above 5.1 mmol according to the International Association of the Diabetes and Pregnancy Study Groups (IADPSG) criteria [29]. Furthermore, fetuses/neonates with the following characteristics were excluded: fetal abnormality detected on antenatal karyotype or ultrasound scans, delivery before 27.0 or later than 42.0 weeks, birth weight below 2.3 kg or larger than 4.5 kg, neonatal hypoglycaemia, fetal death, still birth, and/or neonatal death. We used the 2.3 kg cut-off instead of 2.5 kg because the WHO Multicentre Growth Reference Study Group [30] showed that the first percentile in the boys and girls in the healthy cohort was 2.3 kg. The choice of pre-pregnancy BMI cut-offs took Asian pattern of BMI and metabolic diseases into account [31].

### Measurements

In addition to their first trimester antenatal dating ultrasound scan, participants returned to the hospitals at 19–21, 26–28 and 32–34 weeks of gestation for ultrasound scans, among other antenatal and post-natal assessments. All the ultrasound measurements were measured based on the standard views used in Fetal Medicine Foundation [32]. Fetal head circumference was measured on the transventricular view, which was obtained at the level of the thalami, with visualisation of the falx cerebri and cavum septum pellucidum. Symmetrical appearance of both hemispheres was ensured in the measured section. Abdominal circumference was measured at the level of the stomach and where the umbilical vein was at the level of the portal sinus. For femur diaphysis length measurement, the longest axis of the ossified diaphysis was measured. Machines used in the study were GE Voluson 730 Expert, transabdominal probe (AB2-7, 2-7 MHz broadband curved array transducer) and GE Voluson 730 PRO, transabdominal probe (4CA, broadband curved array transducer).

All the sonographers who participated in the GUSTO study were accredited. They were trained according to standard operating procedures and assessed by the lead sonographer at each site. All the images obtained were standardized with all the necessary landmarks identified as afore-mentioned. Re-assessments were conducted on a regular basis to ensure standardization between the sonographers and across sites. Quality control monitoring of randomly selected images by the sonographers ensured adherence to the protocol and reproducibility. The equipment was calibrated with quality assurance checks by service engineers every six months.

Gestational age (GA) was in exact weeks unless otherwise specified (e.g. 27 weeks and 5 days = 27.7 weeks). GA at first antenatal ultrasound dating scan was estimated by crown-rump length (CRL) if it was not larger than 84 mm [33, 34]. Otherwise, GA at the first scan was estimated by BPD [34, 35]. The estimated fetal weight (in grams) was based on AC, BPD, HC and FL measurements using the Hadlock algorithm [36], log (EFW) = 1*.*3596 − 0*.*00386 × AC × FL + 0*.*0064 × HC + 0*.*00061 × BPD × AC + 0*.*0424 × AC + 0*.*174 × FL. We chose this particular Hadlock formula *a priori* over the others, because it was based on four biometric parameters which may provide a more accurate estimate for the EFW than its counterparts that based on less than four parameters. For gender-specific analyses, gender was based on neonatal assessment.

### Statistical methods

We used the mixed-effect linear regression models for longitudinal data to develop the unconditional and conditional standards [37, 38]. This method accounts for non-normal distribution in the biometric measurements by Box-Cox transformation, captures nonlinear relationship between biometry and GA via a linearizing function of GA obtained by fractional polynomials, and generates z-scores and percentiles by mixed-effects linear modelling of the transformed variables. The method has been used in the development of EFW references for an African [14] and Norwegian population [39]. Details of the methods are available in the aforementioned publications. To facilitate the application of our findings, we provide electronic spreadsheets as Online Digital Supplements, which also provide the formula details in the program codes.

We conducted model diagnostics in three ways. Firstly, we used the detrended Q-Q plot, also known as the worm plot, for visual assessment of the normality of z-scores [40]. Secondly, for each pair of measurements from two consecutive visits, the Pearson’s correlation coefficients between the conditional z-score at the present assessment and the z-score at the previous assessment were calculated. Properly developed standards should give approximately zero correlation. Thirdly, we examined the proportions of observations below the 10th and above the 90th percentiles. The associated 95 % confidence interval for each proportion was obtained via a general linear model for binary outcomes with an identity link. Logistic regression was also used to check that the proportions below (or above) the specified percentiles were independent of predictor(s). For the unconditional standards, the predictor examined was GA. For the conditional standards, the predictors examined were GA and the previous AC or EFW. Properly developed standards should show no association with these predictors. The regression models used the Huber-White robust standard error estimator for statistical inference to adjust for multiple ultrasound scans per participant [38, 41]. All statistical analyses were performed using the Stata statistical software version 12.1 [42].

## Results

### Participant characteristics

There were 626 Chinese women who conceived naturally with singleton pregnancies in the GUSTO study. A total of 312 women were excluded as per the exclusion criteria afore-described. One woman was excluded due to implausible AC measurements during data inspection. The final analysis sample for constructing AC standards consisted of 920 measurements from 313 women, of whom 294 had three and 19 had two ultrasound scans other than the GA dating scan. The analysis sample included 168 male and 145 female foetuses. Inspection of the other biometric measurements was also conducted to identify implausible measurements. This led to exclusion of two more women from the EFW analysis. The final analysis sample for constructing EFW standards consisted of 901 measurements from 311 women, of whom 280 had three and 30 had two EFW measures. The analysis sample included 167 male and 144 female foetuses.

The mean (SD) age, years of formal education, height and pre-pregnancy weight of the women was 31.2 (4.8) years, 13.8 (3.8) years, 159.2 (5.8) cm and 53.0 (6.8) kg, respectively. Mean (SD) of paternal height was 171.3 (6.2) cm. Mean (SD) of GA at dating scan was 12.5 (0.8) weeks. Forty seven per cent were nulliparous. Ninety eight per cent were married or co-habiting. Fifty three per cent had spontaneous labor. The measurements of hemoglobin levels before 15 completed weeks of GA were only available in 176 out of the 313 eligible women, and the mean (SD) hemoglobin levels was 12.5 (1.1) g/dL. Twenty three per cent had caesarian section. All babies were born at full term, with four admitted to neonatal ICU. There was no maternal ICU admission after delivery. Table 1 summarizes the AC and EFW measurements between 18 and 35 completed weeks of GA.

**Table 1:** Summary statistics of the abdominal circumference and estimated fetal weight at each completed gestational week

Completed gestational week	Abdominal circumference (mm)									Estimated fetal weight (gram)								
	Both Genders Pooled			Male			Female			Both Genders Pooled			Male			Female		
	N	Mean	SD	N	Mean	SD	N	Mean	SD	N	Mean	SD	N	Mean	SD	N	Mean	SD
18	10	133	7	8	134	8	2	131	2	9	244	25	7	243	26	2	250	26
19	81	142	7	38	144	7	43	140	7	80	288	26	37	295	29	43	282	23
20	148	153	7	79	154	6	69	151	7	146	344	32	79	348	31	67	340	32
21	57	161	8	35	163	6	22	158	9	55	393	37	33	403	29	22	379	43
22	5	158	7	1	151	--	4	160	7	4	408	43	0	--	--	4	408	43
24	3	201	4	2	202	6	1	199	--	3	748	14	2	743	16	1	758	--
25	17	207	11	8	206	10	9	207	12	16	787	78	7	777	79	9	794	82
26	120	215	10	67	217	9	53	213	10	118	894	83	66	903	84	52	883	81
27	122	223	9	62	225	8	60	221	9	121	1005	98	62	1023	97	59	986	98
28	36	233	11	20	235	10	16	232	12	36	1132	128	20	1139	109	16	1123	151
29	8	245	9	6	248	8	2	238	8	8	1281	105	6	1331	57	2	1130	6
30	3	251	5	1	254	--	2	250	6	2	1452	97	1	1521	--	1	1384	--
31	23	269	12	12	268	15	11	270	8	21	1744	164	10	1715	198	11	1771	130
32	151	279	12	80	280	12	71	278	13	151	1898	189	80	1922	186	71	1871	189
33	116	287	13	63	289	11	53	284	14	112	2063	207	62	2101	192	50	2016	218
34	15	295	10	9	299	7	6	289	12	15	2239	201	9	2308	162	6	2134	223
35	5	305	21	2	288	11	3	316	18	4	2458	429	2	2156	204	2	2761	381

*N* number of measurements, *SD* standard deviation

### Statistical modelling of repeated measurements

For AC, the Box-Cox transformation controlling for GA gave a power transformation parameter estimate $\hat{\lambda}$=0.09 (95 % CI: -0.04 to 0.22) in the analysis pooling both genders, $\hat{\lambda}$=0.03 (95 % CI: -0.16 to 0.22) in males and $\hat{\lambda}$=0.08 (95 % CI: -0.10 to 0.26) in females. The null hypothesis of normal distribution (equivalent to *λ* = 1) was rejected (P < 0.001). All three parameter estimates were near zero with their respective 95 % confidence intervals including zero, suggesting that the natural (base e) logarithmic transformation (equivalent to λ = 0) was suitable for transforming the distribution towards normality [37]. Thus we used log(AC) in the analysis (base e).

The Box-Cox transformation found that a power transformation with a power term -0.1 was appropriate for normalizing EFW: $\hat{\lambda}$=-0.10 (95 % CI: -0.14 to -0.06) for analysis pooling both genders, $\hat{\lambda}$=-0.11 (95 % CI: -0.18 to -0.05) for males and $\hat{\lambda}$=-0.10 (95 % CI: -0.16 to -0.04) for females. Thus we used EFW^− 0.1^ in the analysis.

Results for the linearizing function and the subsequent linear mixed-effect model estimation for transformed AC and EFW are shown in Table 2. In the analysis pooling both genders, the nonlinear relation between log(AC) and GA can be described by a second-degree fractional polynomial model with powers -2 and 1. The systematic component of the model was

**Table 2:** Regression modelling of transformed abdominal circumference and estimated fetal weight in relation to gestational age

	Step	Parameter	Both genders pooled	Male	Female
Transformed AC	Fractional polynomial transformation of GA	*p*_1_	-2	-2	-2
		*p*_2_	1	1	1
		*a*_1_	-168.4982	-164.9962	-172.1061
		*a*_2_	0.0297	0.0297	0.0298
	Mixed-effects model regressing log(AC) on f(GA) = GA^− 2^ + (*a*_2_/*a*_1_)GA	*b*_0_	4.8170	4.8201	4.8114
		*b*_1_	-168.4671	-165.0468	-172.0514
		var(*β*_0*i*_)	0.0012	0.0009	0.0012
		var(*β*_1*i*_)	18.5802	32.9797	0.0999
		cov(*β*_0*i*_, *β*_1*i*_)	0.0490	0.0747	-0.0108
		var(*e*_*ij*_)	0.0008	0.0008	0.0009
Transformed EFW	Fractional polynomial transformation of GA	*p*_1_	0.5	0.5	0.5
		*p*_2_	2	2	2
		*a*_1_	-0.1176268	-0.1166731	-0.1186321
		*a*_2_	0.0796 × 10^-3^	0.0783 × 10^-3^	0.0809 × 10^-3^
	Mixed-effects model regressing EFW^-0.1^ on f(GA) = GA^0.5^ + (*a*_2_/*a*_1_)GA^2^	*b*_0_	1.0573	1.0528	1.0623
		*b*_1_	-0.1176	-0.1167	-0.1186
		var(*β*_0*i*_)	7.370 × 10^-6^	3.004 × 10^-4^	16.70 × 10^-6^
		var(*β*_1*i*_)	1.668 × 10^-4^	14.2 × 10^-6^	7.74 × 10^-12^
		cov(*β*_0*i*_, *β*_1*i*_)	-0.335 × 10^-4^	-0.64 × 10^-4^	-1.12 × 10^-8^
		var(*e*_*ij*_)	6.290 × 10^-6^	5.43 × 10^-6^	7.13 × 10^-6^

*AC* abdominal circumference, *EFW* estimated fetal weight, *GA* gestational age in exact weeks

$$ \log \left(\mathrm{AC}\right)=-168.4982\times {\mathrm{GA}}^{-2}+0.0297\times \mathrm{G}\mathrm{A}=-168.4982\times f\left(\mathrm{G}\mathrm{A}\right) $$

where *f*(GA) = GA^− 2^ − 0.0297/168.4982 × GA was the linearizing function. The transformed GA variable, f(GA), linearized the non-linear relation between log(AC) and GA. Upon fitting a mixed-effects regression of log(AC) on the transformed GA variable f(GA), the estimated mean of log(AC) is given by (model details in Table 2):

$$ \log \left(\mathrm{AC}\right)=4.8170-168.4671\times f\left(\mathrm{G}\mathrm{A}\right). $$

The analysis results for the (power-transformed) EFW measurements are also given in Table 2. They can be interpreted in the same way as above for log(AC).

### Unconditional standards

**Fig. 1:**
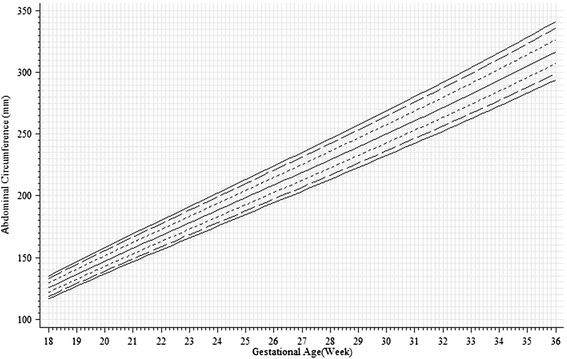
Unconditional chart for abdominal circumference pooling both genders (solid lines: 5th, 50th and 95th percentiles; long dashed lines: 10th and 90th percentiles; short dashed lines: 25th and 75th percentiles). Tick marks at multiples of 5 mm from 100 to 350 mm on the vertical axis, and at single day on the horizontal axis

**Fig. 2:**
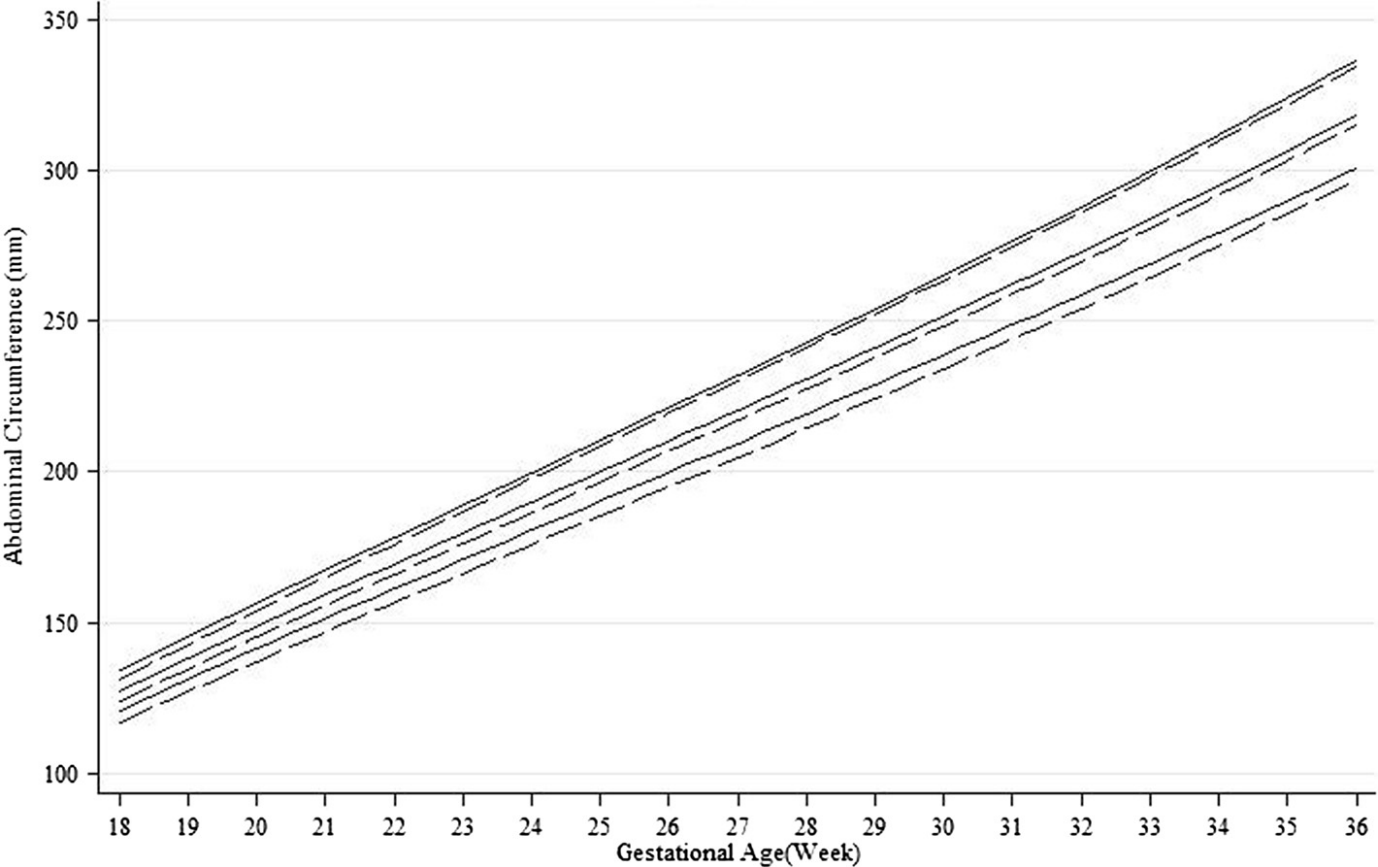
Unconditional gender-specific 10th, 50th and 90th percentiles for abdominal circumference (solid lines for males and broken lines for females)

The unconditional z-scores and percentiles can be obtained by plugging the information in Table 2 into the formula in Royston [37] (also in Landis *et al.* [14], Cheung [38], Johnsen *et al.* [39]). Details of the calculations and details of all the unconditional charts discussed can be found in the supplied electronic spreadsheet UnconditionalChart_FetalGrowth_Supp.xls (see Additional file 1). The unconditional standard for both genders pooled is shown in Fig. 1 in terms of percentiles, after back-transforming the AC and GA values to their original scales. Each percentile increased roughly linearly with GA from 18 to 36 exact weeks. The vertical distance between percentiles increased over time, indicating increase in variability as fetuses grew. Similar patterns were observed in both gender-specific charts (Fig. 2). The median AC values for males were about 1 % to 2 % larger than those for females at the same GA. The 10th and 90th percentile values for males were about 2 % and 1 %, respectively, higher than those for females at the same GA.

**Fig. 3:**
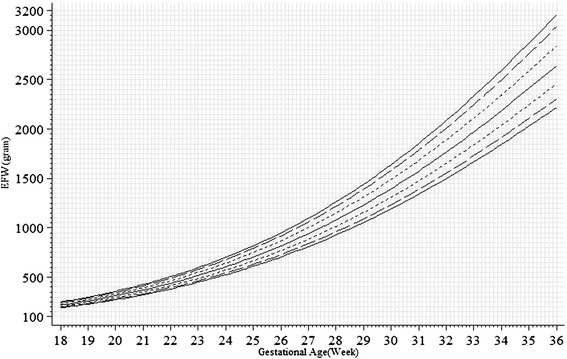
Unconditional chart for estimated fetal weight (EFW) pooling both genders (solid lines: 5th, 50th and 95th percentiles; long dashed lines: 10th and 90th percentiles; short dashed lines: 25th and 75th percentiles). Tick marks at multiples of 50 grams from 100 to 3200 grams on the vertical axis, and at single day on the horizontal axis

**Fig. 4:**
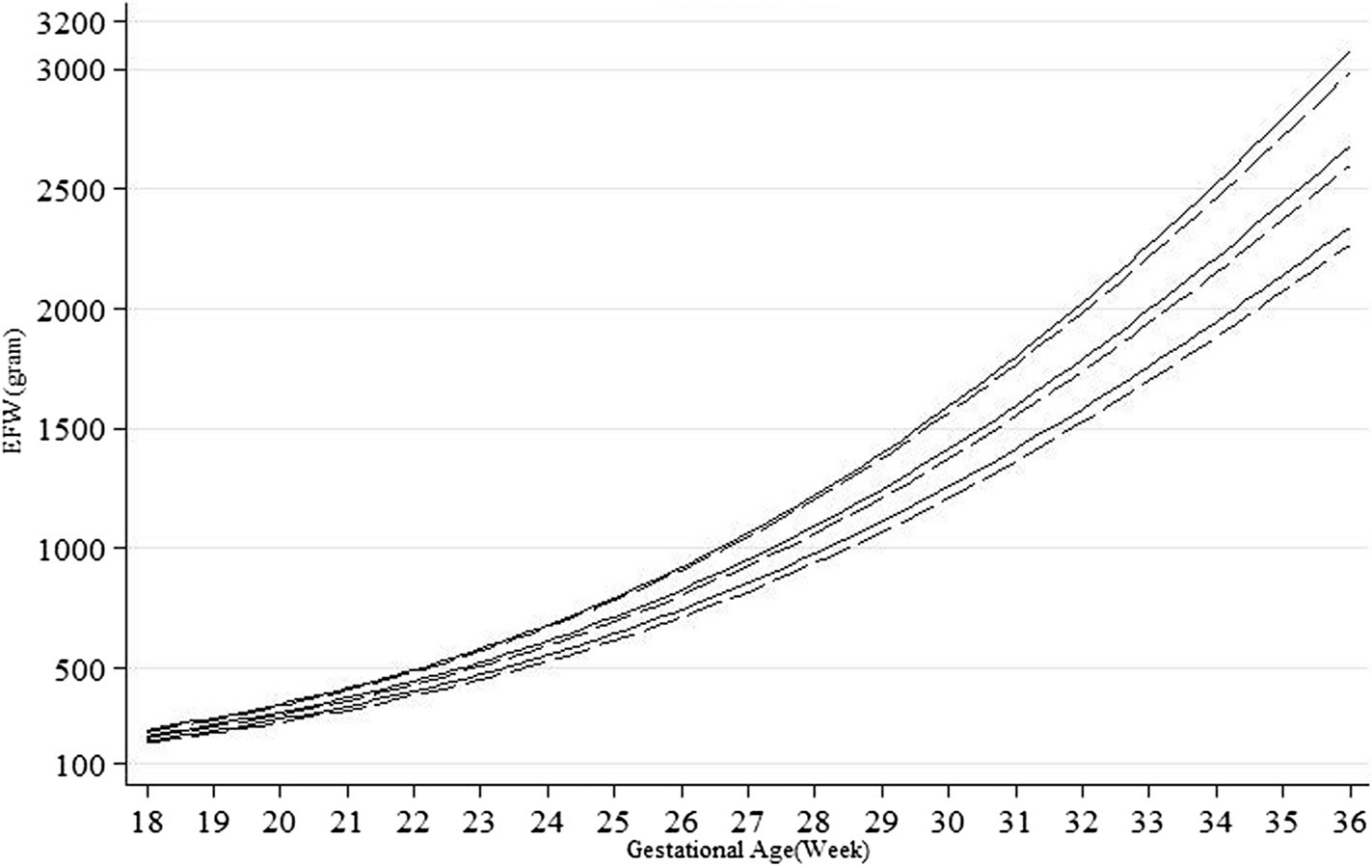
Unconditional gender-specific 10th, 50th and 90th percentiles for estimated fetal weight (EFW) (solid lines for males and broken lines for females)

Unconditional standards for EFW pooling both genders and specific to each gender were shown in Figs. 3 and 4. The gain in EFW showed acceleration as GA increased within from 18 to 36 weeks. Similar to AC, there was more gender-difference in the 10th percentile than in the 50th or 90th percentiles. The gender differences in grams were similar between the 10th, 50th and 90th percentiles. In terms of percentage, however, the differences in the 10th, 50th and 90th percentile were approximately 4 %, 3 % and 2 %, respectively.

### Conditional standards

**Fig. 5:**
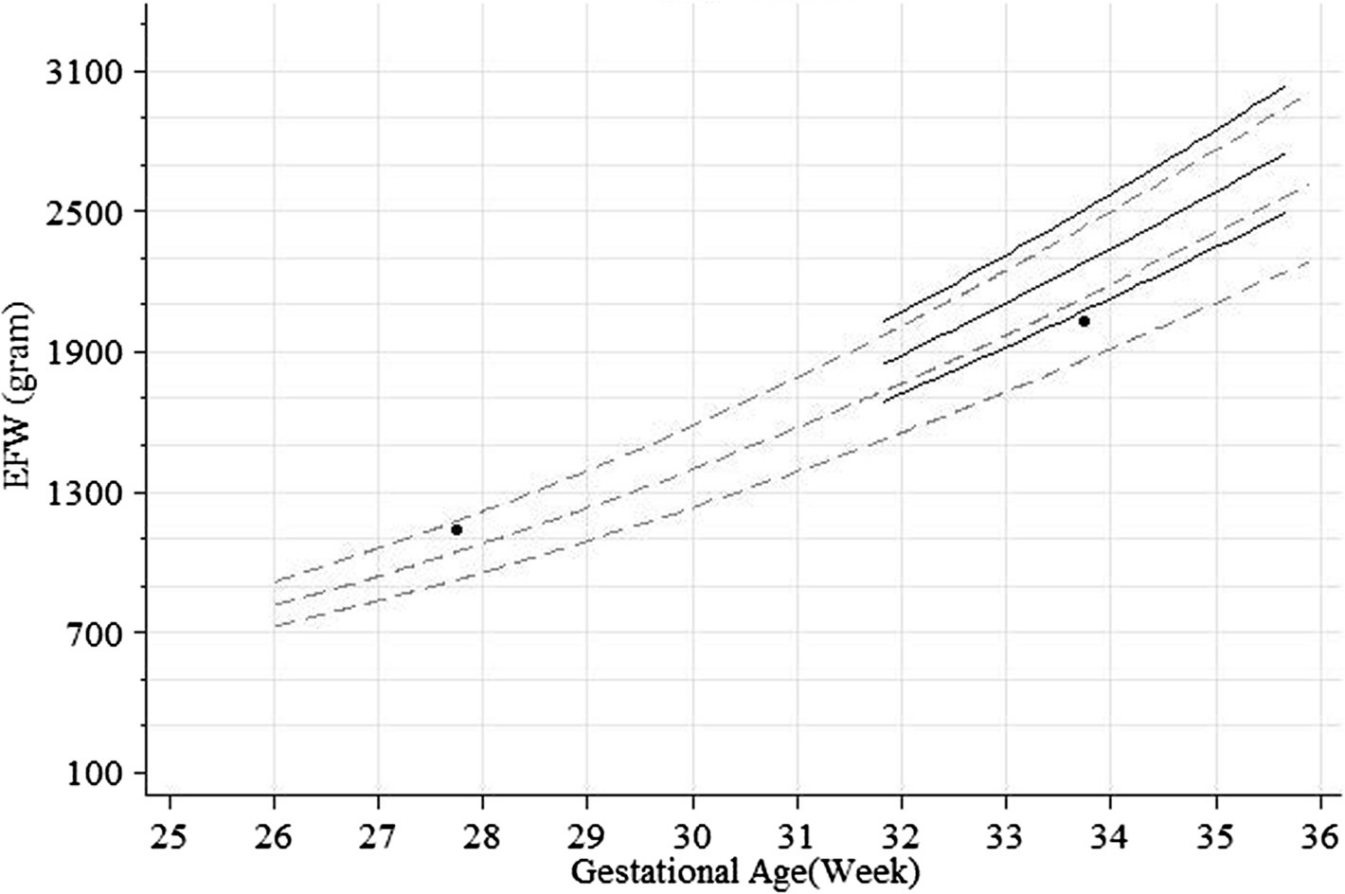
Conditional versus unconditional EFW standards for participant ID “020-66086”: a foetus (●) whose EFW was 1138 grams at gestational age of 27.7 weeks (i.e. 27 weeks + 5 days) and 2024 grams at gestational age of 33.7 weeks (i.e. 33 weeks + 5 days). (Broken lines from bottom to top: unconditional 10th, 50th and 90th percentiles. Solid lines from bottom to top: conditional 10th, 50th and 90th percentiles)

The conditional z-scores and percentiles can be obtained by plugging the information in Table 2 into the formula in Royston [37] (also in Landis *et al.* [14], Cheung [38], Johnsen *et al.* [39]), which has also been implemented in the supplied electronic spreadsheet ConditionalChart_FetalGrowth_Supp.xlsx (see Additional file 2). To illustrate, participant number 020-66086 had EFW 1138 grams at gestational age 27.7 and 2024 grams at 33.7 weeks (Fig. 5). Using the unconditional standards, the second assessment would be considered normal as it fell above the unconditional 10th percentile (z-score = -0.46). However, using the conditional standards to take into account the previous EFW measurement, the present EFW fell below the conditional 10th percentile (z-score = -1.62).

### Model diagnostics

The detrended Q-Q plot for the unconditional standard for AC, pooling both genders showed satisfactory fit (Additional file 3: Figure S1). Most of the data points were around the horizontal lines, which indicate normal distribution of the z-scores. Only six of the 920 z-scores fell outside the 95 % confidence interval (CI). The detrended Q-Q plot of the conditional z-scores also showed satisfactory fit (Additional file 4: Figure S2). Only eight of the 607 z-scores fell outside the 95 % CI. The correlation coefficients between the conditional z-scores and the previous (unconditional) z-scores for each pair of two consecutive study visits (i.e. between 19-21 and 26-28 weeks, and between 26-28 and 32-34 weeks) were both close to zero: -0.06 (P = 0.319) and 0.03 (P = 0.656), respectively. The proportions of the 920 measurements classified below the 10th percentile and above the 90th percentile of the unconditional standards pooling both genders were 10.9 % (95 % CI: 8.4 % to 13.4 %) and 9.7 % (95 % CI: 7.5 % to 11.8 %), respectively. Furthermore, logistic regression showed no association between gestational age and the classifications below the 10th or above the 90th percentile (each P > 0.1). Similarly, the proportions classified below the 10th and above the 90th percentiles of the conditional standards were 10.2 % (95 % CI 7.8-12.6 %) and 8.6 % (95 % CI 6.4-10.7 %), respectively. None of the classifications was associated with either gestational age or the previous AC measurement (each P > 0.1). Results on model diagnostics for the gender-specific unconditional and conditional standards were similar and both demonstrated satisfactory performance (details not shown).

The detrended Q-Q plots of unconditional and conditional EFW standards indicated sufficient fit, with the data points mostly scattered around the horizontal lines (Additional file 5: Figures S3; Additional file 6: Figures S4). Only ten of the 901 unconditional z-scores and seven of the 590 conditional z-scores fell outside the 95 % CIs. The correlation coefficients between the conditional z-scores and the initial (unconditional) z-scores for each pair of two consecutive study visits were both close to zero: 0.05 (P = 0.358) and -0.05 (P = 0.355). The proportions of the 901 EFW measurements classified below 10th percentile or above the 90th percentile of the unconditional standards pooling both genders were 9.2 % (95 % CI: 6.7 % to 11.7 %) and 10.4 % (95 % CI: 7.9 % to 12.9 %), respectively. Logistic regression showed no association between gestational age and the classifications (each P > 0.1). Similarly, the proportions classified below the 10th and above the 90th percentiles of the conditional standards were, respectively, 10.3 % (95 % CI: 7.9 % to 12.7 %) and 9.5 % (95 % CI: 7.2 % to 11.7 %). None of the classifications was associated with gestational age or previous EFW measurement (each P > 0.1). Results on model diagnostics for the gender-specific unconditional and conditional standards were similar and both demonstrated satisfactory performance (details not shown).

## Discussion

We have developed standards of fetal abdominal circumference and estimated fetal weight for ethnic Chinese in Singapore. This analysis included a healthy cohort according to the characteristics of the women, pregnancies and birth outcomes. This results in a set of growth standards, as opposed to growth references [20, 30, 43, 44]. A reference describes what happens in the general population and is descriptive in nature. A standard describes what should happen in a “healthy” population and is prescriptive. Such distinction was also discussed in Bertino *et al.* [43]. The World Health Organization’s Multi-Center Growth Reference Study was explicitly a project to develop standards, which is considered more appropriate for growth monitoring [30]. Similar efforts have been followed by the INTERGROWTH-21st Project to develop fetal, preterm, and neonatal standards [20, 44]. In contrast to some previous studies, we have provided not only the unconditional standards but also the conditional standards. The statistical methods we used are parametric analysis (of transformed variables). Recent methodological exploration has demonstrated that the resultant percentiles are robust unless there are serious violations of model assumptions [45]. Currently there is a shortage of non-parametric methods for longitudinal data in this context. We conducted and reported various aspects of model diagnostic, showing sufficient goodness-of-fit.

In this study, we estimated gestational age based on ultrasound measurement instead of last menstrual period (LMP) for three reasons. Firstly, ultrasound dating in the first trimester is the most accurate method to estimate gestational age. If LMP and first trimester ultrasound estimates are both available but they disagree, clinical guidelines recommend to use the ultrasound estimate [46, 47]. Secondly, there were about 10 % of study participants who did not provide information on their last menstrual periods. If the LMP method was to be used for constructing the growth standards, the sample size would have been 10 % less than the current sample size. Furthermore, the non-availability of LMP data in this study also reflects that in clinical practice it is difficult to rely on LMP data for gestational age estimation. Thirdly, the use of ultrasound estimate in constructing fetal growth standards agrees with recommended clinical practice [46, 47]. As such, our research outputs are useful for clinical and research practices based on ultrasound. In countries that primarily used LMP method and for studies that focus on natural biological variability, our results may not be applicable.

Clinical experience may alert a physician that a large degree of percentile crossing on an unconditional growth chart warrants further investigation. The use of conditional growth charts formalizes and quantifies this assessment of change over time. It has been suggested that conditional standards are more sensitive in fetal growth monitoring [25]. This hypothesis and the practical utility of conditional standards have not been sufficiently assessed, as conditional standards are rarely available to begin with. The present development will facilitate not only fetal growth monitoring in Singapore but also further research along this line. That said, we consider the two sets of standards complementary, not competitive. It is likely that unconditional standards are more useful in identifying chronic problems whereas conditional standards are more sensitive in identifying acute problems [24].

A limitation of the present study is that the sample size is relatively small for studying adverse birth outcomes in a low mortality/morbidity setting. Therefore we have only produced the AC and EFW unconditional and conditional standards, without comparing their performance in detecting adverse birth outcomes*.* Another limitation of the study is that the ultrasound measurements were not evenly distributed over the range of GA, with concentration around 19-21, 26-27 and 32-33 weeks. So, the accuracy of the standards has not been fully assessed at each GA week. However, these are typical timing of antenatal visits. The standards are sufficient and accurate at least for practical use and research based on typical antenatal visit schedules. Further research that involves larger sample size for analysis of adverse birth outcomes and more observations evenly distributed over a larger range of GA are warranted.

We compared this set of growth standards versus other previously developed references/standards. As the underlying research methods vary across the studies, we only highlight the important similarities and differences between previous findings and ours. In terms of fetal AC, comparing to other ethnic-specific norms, our standards had the same median of 188 mm at 24 weeks gestation as a previous Singapore reference [48] and a Chinese standard from the Central-South China Fetal Growth Study [49] and was similar to the 191 mm in the INTERGROWTH-21st Project [44]. By a late gestation of 36 weeks, however, the median fetal AC in the present standards was 317 mm, surpassing the median of 303 mm in the previous Singapore reference and the median of 301 mm in the Chinese standards. This median fetal AC was, in fact, similar to more recent, non-Chinese norms, including the norms derived in Korea (312 mm) [18], Pakistan (314 mm) [50], Peru (315 mm) [12], and the INTERGRWOTH-21st Project (312 mm) [44] and approached the median of 329 mm in a Caucasian-only cohort in London, UK [51]. In contrast, the median in a set of Hong Kong Chinese standards is consistently higher than the present median from 24 to 36 weeks [52].

Our EFW standards also showed a similar trajectory of starting low at early gestations and then, catching up at later gestations. At 20 weeks gestation, our healthy cohort’s median EFW of 312 g was almost identical to 315 g in a sub-Saharan African reference population [14]. The latter rapidly fell behind with advancing gestation, as compared to Western cohorts from the UK [51] and Norway [39]. In contrast, our EFW standards had a lower median EFW of 606 g at 24 weeks gestation and caught up to 2644 g at 36 weeks gestation, as compared to a contemporary French reference of 662 g at 24 weeks gestation and 2624 g at 36 weeks gestation [53]. This is confirmed by using the “global” reference developed by Mikolajczyk *et al.* which generated a median of 651 g at 24 weeks gestation and 2619 g at 36 weeks gestation [21]. The use of global or foreign norms to characterize fetal growth, both AC and EFW, in late gestation appears to be robust. However, for the characterization earlier in gestation, which is important for early detection of fetal growth restriction, global or foreign norms are not accurate.

## Conclusion

In summary, we have developed unconditional and conditional standards for monitoring fetal abdominal circumference and estimated fetal weight from 18 to 36 weeks of gestation in ethnic-Chinese population. As compared to other standards, the local standards indicate growth pattern that are starting low at early gestations and catching up at later gestations.

## Additional files

Additional file 1:
**Electronic spreadsheet UnconditionalChart_FetalGrowth_Supp.xlsx.**
Additional file 2:
**Electronic spreadsheet ConditionalChart_FetalGrowth_Supp.xlsx.**
Additional file 3: Figure S1.
**Detrended Q-Q plot for the unconditional standard for AC, pooling both genders.**
Additional file 4: Figure S2.
**Detrended Q-Q plot for the conditional standard for AC, pooling both genders.**
Additional file 5: Figure S3.
**Detrended Q-Q plot for the unconditional standard for EFW, pooling both genders.**
Additional file 6: Figure S4.
**Detrended Q-Q plot for the conditional standard for EFW, pooling both genders.**
