# Supplementary figures and images for: Unconditional and conditional standards for fetal abdominal circumference and estimated fetal weight in an ethnic Chinese population: a birth cohort study
Source: BMC Pregnancy Childbirth. 2015 Jun 25;15:141. doi: 10.1186/s12884-015-0569-1 (PMC4480986; doi:10.1186/s12884-015-0569-1)

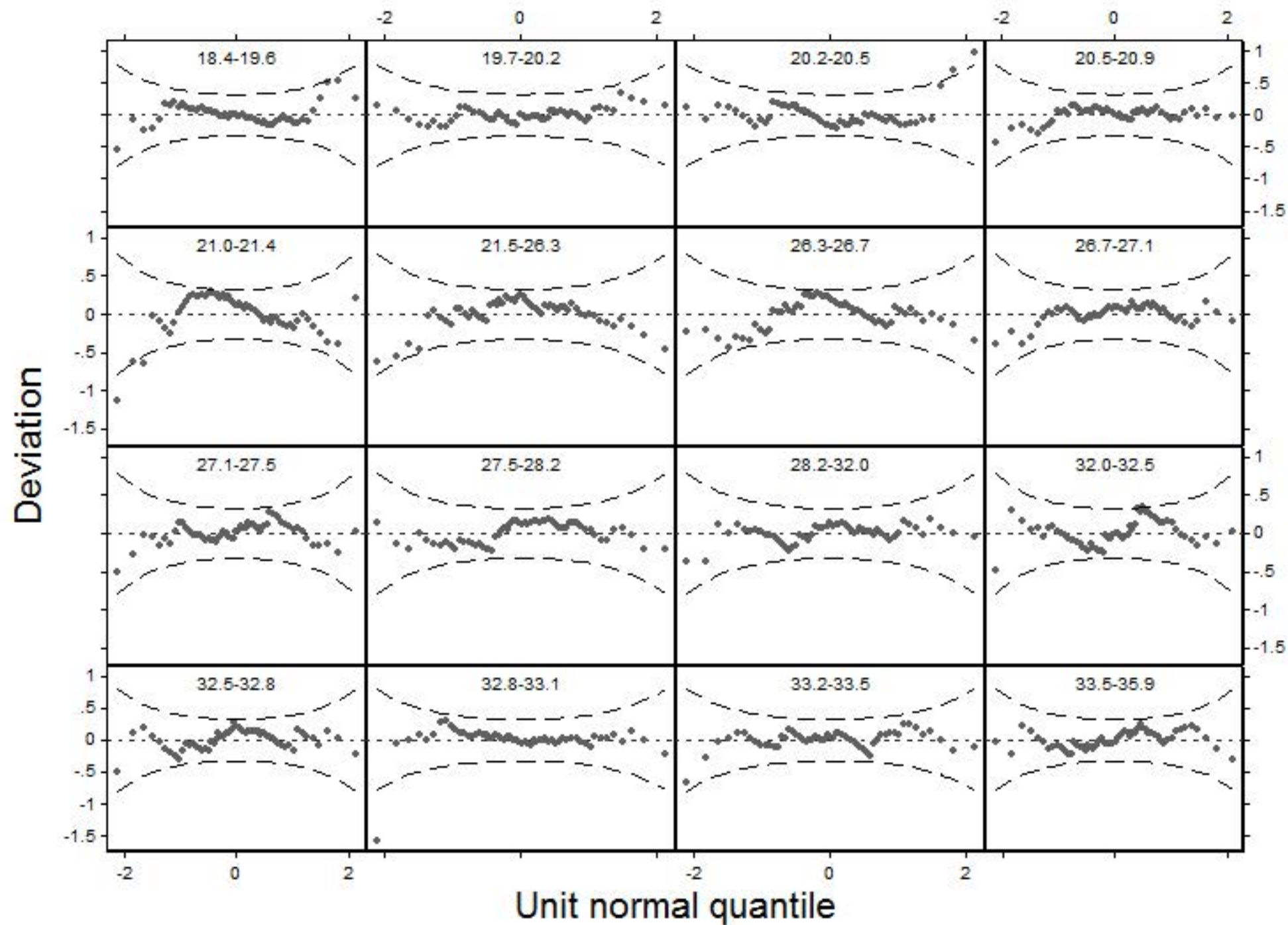

Supplement: Additional file 3: Figure S1. — Detrended Q-Q plot for the unconditional standard for AC, pooling both genders. [file 12884_2015_569_MOESM3_ESM.pdf]

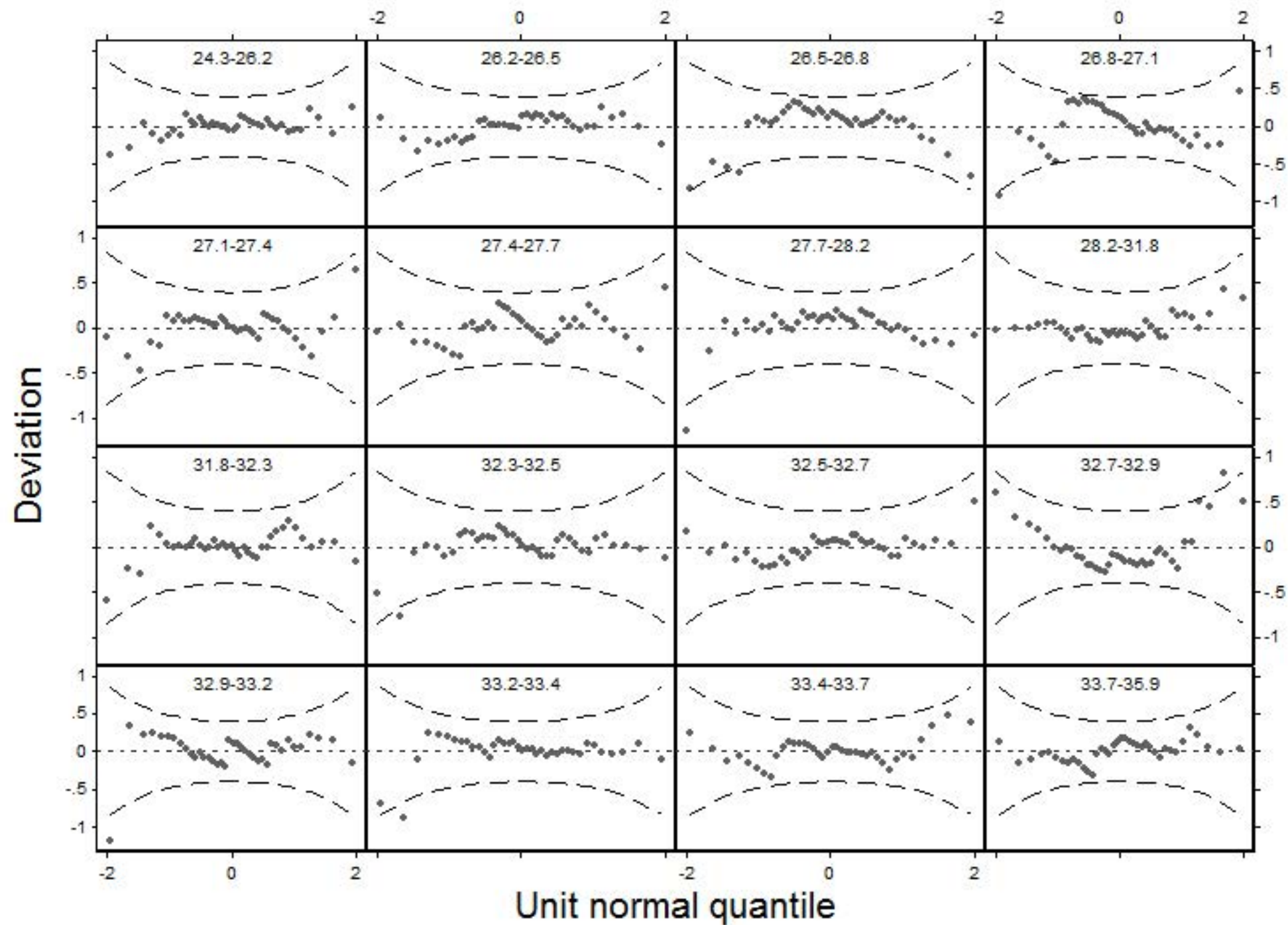

Supplement: Additional file 4: Figure S2. — Detrended Q-Q plot for the conditional standard for AC, pooling both genders. [file 12884_2015_569_MOESM4_ESM.pdf]

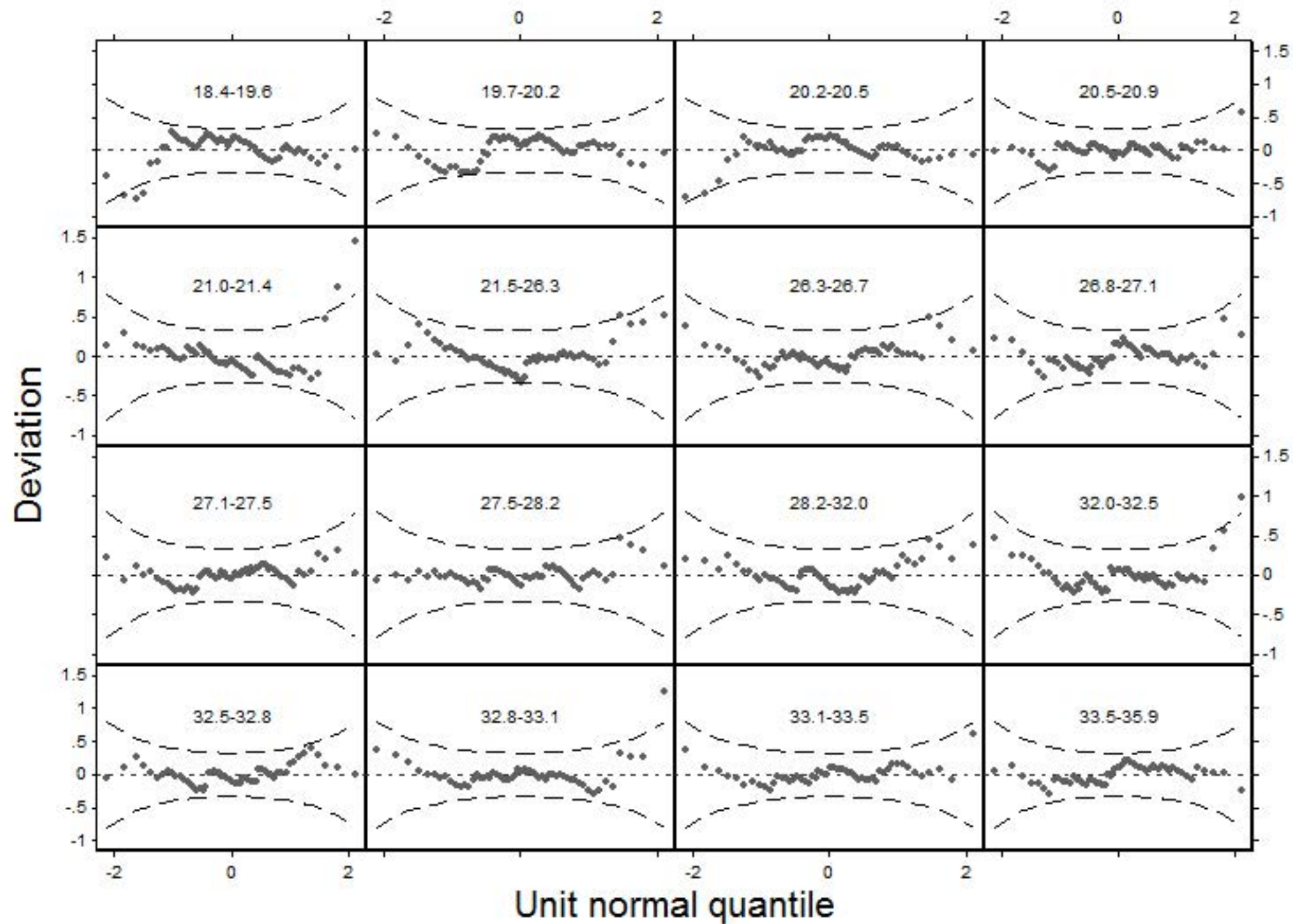

Supplement: Additional file 5: Figure S3. — Detrended Q-Q plot for the unconditional standard for EFW, pooling both genders. [file 12884_2015_569_MOESM5_ESM.pdf]

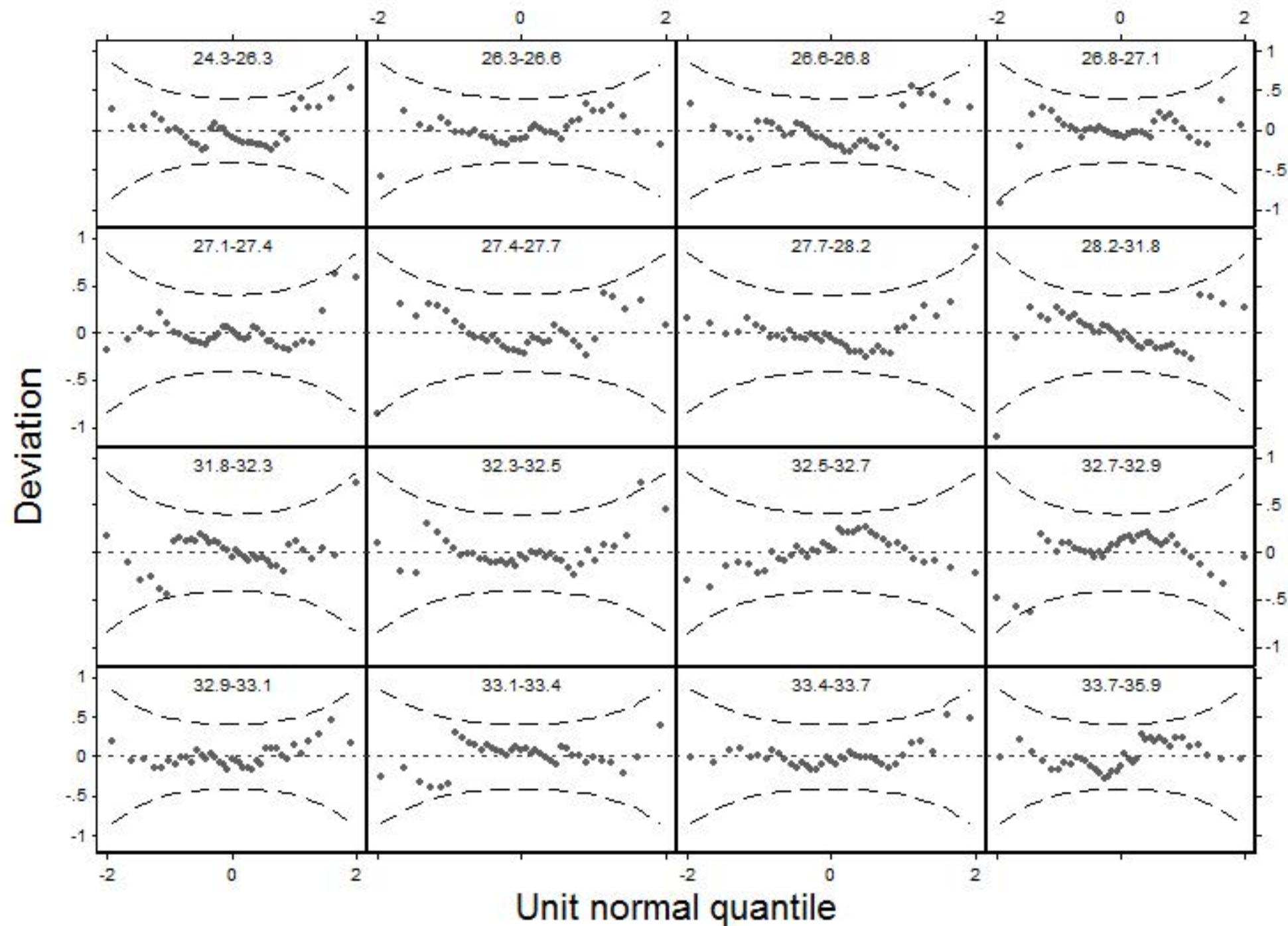

Supplement: Additional file 6: Figure S4. — Detrended Q-Q plot for the conditional standard for EFW, pooling both genders. [file 12884_2015_569_MOESM6_ESM.pdf]
